# Finger Vein Recognition Based on a Personalized Best Bit Map

**DOI:** 10.3390/s120201738

**Published:** 2012-02-09

**Authors:** Gongping Yang, Xiaoming Xi, Yilong Yin

**Affiliations:** School of Computer Science and Technology, Shandong University, Jinan 250101, China; E-Mails: gpyang@sdu.edu.cn (G.Y.); fyzq10@126.com (X.X.)

**Keywords:** finger vein recognition, personalized best bit map, local binary pattern, Hamming distance, general framework

## Abstract

Finger vein patterns have recently been recognized as an effective biometric identifier. In this paper, we propose a finger vein recognition method based on a personalized best bit map (PBBM). Our method is rooted in a local binary pattern based method and then inclined to use the best bits only for matching. We first present the concept of PBBM and the generating algorithm. Then we propose the finger vein recognition framework, which consists of preprocessing, feature extraction, and matching. Finally, we design extensive experiments to evaluate the effectiveness of our proposal. Experimental results show that PBBM achieves not only better performance, but also high robustness and reliability. In addition, PBBM can be used as a general framework for binary pattern based recognition.

## 1. Introduction

Biometric recognition, or simply biometrics, refers to the use of distinctive anatomical and behavioral characteristics or identifiers (e.g., fingerprints, faces, iris, voices, and hand geometries) for automatically recognizing an individual [1,2]. Since it is difficult to misplace, forge or share biometric identifiers, biometric recognition is more reliable than traditional token-based methods (e.g., keys or ID cards) and knowledge-based methods (e.g., passwords or PINs). Besides, biometric recognition also promises better security, higher efficiency, and better user experience in many cases. Due to these advantages, biometric recognition systems have been extensively deployed and employed in a large number of government agencies (e.g., border crossing, national ID cards, e-passports, *etc*.) and daily applications (e.g., computer network logons, mobile phones, Web access, smartcards, *etc*.).

Hand-based biometrics normally include fingerprint recognition, finger knuckle print recognition, and palm print recognition, among which fingerprint recognition is the most mature method and has been used in many fields for years [2]. However, fingerprint based biometric system are vulnerable to forgery because fingerprints are easily exposed to others. What is even worse is that a finger’s surface conditions (such as sweat and dryness) can significantly deteriorate the acquisition of clear fingerprint patterns, resulting in a dramatically degraded performance [3]. Finger knuckle print [4] and palm print [5] based biometric systems are easy to replicate since the features are external to human bodies.

To overcome the limitations of the current hand-based biometric systems, finger vein recognition was proposed and has been well studied recently [6]. In [7], the authors prove that each finger has a unique vein pattern that can be used for personal verification. The finger vein recognition has some advantages over other hand-based biometric authentication techniques [8,9]: (1) *non-contact*: finger vein patterns are not influenced by surface conditions. Non-invasive and contactless data capture ensures both convenience and cleanliness for the users, and it is more acceptable for the users; (2) *live body identification*: finger vein patterns can only be identified on a live body; (3) *high security*: finger vein patterns are internal features that are difficult to forge; (4) *small device size*: as compared to palm vein based verification devices [10], most finger vein recognition devices are smaller in size.

Like other traits, finger vein recognition includes four main steps, including image capturing, pre-processing, feature extraction and matching. In order to better utilize the features from the segmented blood vessel network for recognition, [6] extracts the finger vein pattern from the unclear image with line tracking, which starts from various positions. In [11], the minutiae features include bifurcation points and ending points are extracted from these vein patterns. These feature points are used for geometric representation of the vein patterns’ shape. A modified Hausdorff distance algorithm is provided to evaluate the identification ability among all possible relative positions of the vein patterns’ shape. In [12] the authors propose a mean curvature method, which regards the vein image as a geometric shape and finds the valley-like structures with negative mean curvatures; an equal error rate of 0.25% is reported with test images from 125 fingers. In [13] the patterns of finger veins are extracted by combining two segmentation methods, including Morphological Operation and Maximum Curvature Points in Image Profiles. [14] introduces a wide line detector for feature extraction, which can obtain precise width information for the veins and increases the information of the extracted features from low quality images. Due to the optical blurring and skin scattering problems, the finger vein images are not always clear and can show irregular shadings [15]. To deal with this problem, many finger vein image enhancement methods are proposed [16–20]. A detailed description of these approaches is beyond the scope of this paper. However, a summary of these approaches with the typical references is provided in Table 1.

In order to further boost the performance of finger vein recognition, multi-biometric systems are provided. The example in [21] exploits finger vein features in local moments, topological structure and statistics, respectively, and a fusion scheme is adopted for decision making, obtaining a higher recognition rate of 99% while the FAR is only 0.0086%. The authors in [22] first use a 2-D Gabor filter to filter the original finger vein images, and then extract the phase and direction texture features for combination in a feature level fusion. Finally a modified Hamming distance measure is used for matching. A recognition method based on the score-level fusion of finger veins, fingerprints, and finger geometry features is proposed in [23]. The fusion at the feature extraction level for fingerprints and finger vein biometrics is studied in [24]. The authors also use a dynamic weighting matching algorithm based on quality evaluation of interest features. A multimodal biometric approach fusing finger vein patterns with finger-dorsa textures is introduced in [25], where the binary vein patterns and normalized dorsal textures are fused into one feature image, and a block-based texture feature is proposed for personal authentication.

Most finger vein recognition methods utilize features from the segmented blood vessel network [26]. However, segmentation errors may occur during the feature extraction process due to the low quality of finger vein images. Improperly segmented networks may degrade the recognition accuracy significantly. To solve this problem, binary pattern based methods are proposed [26,27], whereby the captured finger vein images are enhanced by modified Gaussian high-pass filter and then LBP, LDP [27] or LLBP [26] are applied to extract the binary codes from the enhanced images. The similarity between the extracted and enrolled binary codes is measured by Hamming distance. In [26], experimental results on the images from 204 fingers indicated that the equal error rate (3.729%) for the LLBP is significantly lower than that of the LBP and LDP approaches.

When we evaluated LBP based finger vein recognition, we noticed two interesting phenomena: (1) generally speaking, there are many samples captured from the same finger and LBP binary codes (abbreviated in LBPCodes) extracted from these samples, respectively. Comparing each bit location of the LBPCode series, we find most of them are consistent, *i.e.*, the bit values are all 1’s or 0’s. Only a few bits are inconsistent, where some LBPCode series take the value of 1’s, and the others value 0’s. (2) Furthermore, the consistent bits of one finger vein are different from the others in terms of location and bit value. These phenomena motivated us to use these consistent bits for personalized matching. Inspired by [28,29], we propose a finger vein recognition method based on a personalized best bit map (PBBM). Extensive experiments show that PBBM can significantly improve recognition performance as well as robustness.

The rest of this paper is organized as follows: Section 2 presents the principles of LBP-based finger vein recognition. Section 3 introduces the generation of the PBBM. Section 4 describes the framework of finger vein recognition based on a PBBM. Section 5 presents extensive experiments and discusses the robustness of the PBBM approach. Finally, Section 6 concludes this paper.

## 2. LBP Based Finger-Vein Recognition

**Figure 1.**
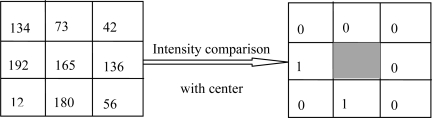
Example of an LBP operator.

Local Binary Pattern (LBP) is a type of feature extraction method. In [30,31] an LBP operator as a nonparametric 3 × 3 kernel for texture classification was proposed. In [27] a LBP-based finger vein recognition method was proposed. An LBP can be defined as an ordered set of binary values determined by comparing the gray values of a center pixel and its neighboring pixels, as shown in Figure 1 The binary values can be expressed in decimal form as shown in Equation (1) [27]:

(1) $$\text{LBP}(x_{c},y_{c})=\sum_{n=0}^{n=7}s(i_{n}-i_{c})2^{n}$$

where i_c_ and i_n_ in Equation (1) denote the gray value of the center pixel (x_c_,y_c_) and its eight neighboring pixels, respectively. The function s(x) is defined as [27]:

(2) $$s(x)=\{\begin{array}{l} 1\;\text{if x}\geq 0\\ 0\;\text{if x}<0 \end{array}$$

Through Equations (1) and (2), the LBP operator extracts a finger vein binary code of M × N × 8 bits, M × N is the size of the image. In Figure 1, the binary sequence on the 3 × 3 block is defined clockwise from the top-left as 00000101.

Denoting the LBP feature of image in the database by LBPCodeA and the LBP feature of input image by LBPCodeB, the hamming distance (HD) is generally adopted to measure dissimilarities between two binary patterns, which are represented as follows:

(3) $$\text{HD}=\frac{\left\|\text{LBPCodeA}⊗\text{LBPCodeB}\right\|}{\text{Code Length}}$$

In Equation (3), ⊗ is a Boolean exclusive-OR operator between two binary patterns.

## 3. Personalized Best Bit Map

**Figure 2.**
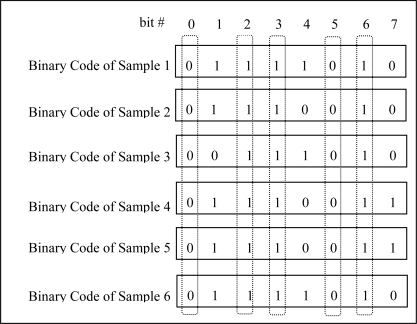
Examples of binary code.

Generally speaking, many samples can be captured from an individual, and we can get corresponding LBPCodes through extracting LBP features from each sample. When investigating these LBPCodes of samples from the same finger vein, a possibly valuable phenomenon attracted our attention: the values of the bits in same location of LBPCodes have different traits. The values of some bits are consistent, either 1’s or 0’s; the values of some bits have majority of 1’s or 0’s; the values of some bits interlace 1’s and 0’s. Figure 2 gives an example of Binary Codes of six samples from a certain individual. Apparently, bit0, bit2, bit3, bit5, bit6 are very consistent, the values of bit1 are mostly 1’s, the values of bit7 are mainly 0’s, and bit4 has interlaced 1’s and 0’s values.

In an ideal situation, if many samples are captured from the same individual, the values of each bit in same location of LBPCodes should be all identical without considering the interference factors such as displacement and rotation. If a bit has different value, we can consider it a noisy bit, which may have a negative effect on recognition performance.

These investigations provide a valuable clue to improve finger vein recognition performance. We can acquire these consistent bits through samples from same individual, record their values and locations in LBPCode. By doing this, when verifying a test sample, we just compare the values of bits at the same location as consistent bits. This may result in better performance in recognition and time consumption than comparing all bits.

Suppose that individual A has *m* samples denoted by Img_1_, Img_2_….Img_m_, the corresponding Binary Codes extracted by the LBP operator are denoted by LBPCode_1_, LBPCode2…LBPCode_m_, the length of Binary Code is *n*, the value of a certain bit *i* is 1’s for *m_1_* times, and 0’s for *m_0_* times (*m_1_* + *m_0_* = *m*), so the stability of the bit is measured as:

(4) $$s_{i}=\frac{\left|m1-m0\right|}{m1+m0}$$

**Definition 1:** If the s*_i_* value of a certain bit is equal to 1, this bit is called Best Bit.
**Definition 2:** All Best Bits of a certain individual make up his Best Bit Map (abbreviated as BBM).

Each Best Bit consists of two components: location and value, where the location means in which LBPCode the Best Bit is located, and the value is assigned as 0’s or 1’s. Because the BBM is different from individual to individual, we call the BBM for each individual the Personalized Best Bit Map (abbreviated as PBBM).

The samples may have some misalignment during the capture process. In order to generate an ideal BBM, the alignment should be considered. Take generating the BBM of two samples as an example. One sample is a reference, and the other sample is aligned through moving toward up, down, left and right direction. Firstly we extract the subsets of the two LBPCodes according to the overlapped region, respectively. Then we investigate the similarity of these two subsets. Finally we use the subsets which have maximum similarity to generate the BBM of the two samples according to definition 1 and 2. The pseudo-code of generating BBM of two samples is summarized in Algorithm 1.

**Algorithm 1.** GetBBM_twosamples.

**INPUT:** LBPCode1 of Img1, LBPCode2 of Img2, (moving) *step*, (moving) *times*.
**OUTPUT:** BBM of two samples Img1 and Img2.
**Algorithm:**
Direct matching, compute the similarity using LBPCode1and LBPCode2, denoted as S0.
//taking Img1as base, align through moving Img2 toward up direction.
for n=1 to *times* do
move Img2 up n*step pixels.
extract the subsets of LBPCode1 and LBPCode2 according overlapped region.
Compute the similarity using these two subsets, denoted as Sup(n).
endfor
//taking Img1as base, align through moving Img2 toward down direction.
for n=1 to *times* do
move Img2 down n*step pixels.
extract the subsets of LBPCode1 and LBPCode2 according overlapped region.
Compute the similarity using these two subsets, denoted as Sdown(n).
endfor
//taking Img1as base, align through moving Img2 toward left direction.
for n=1 to *times* do
move Img2 left n*step pixels.
extract the subsets of LBPCode1 and LBPCode2 according overlapped region.
Compute the similarity using these two subsets, denoted as Sleft(n).
endfor
//taking Img1as base, align through moving Img2 toward right direction.
for n=1 to *times* do
move Img2 right n*step pixels.
extract the subsets of LBPCode1 and LBPCode2 according overlapped region.
Compute the similarity using these two subsets, denoted as Sright(n).
endfor
S=Max(S0,Sup(1)…Sup(times), Sdown(1)…Sdown(times), Sleft(1)…Sleft(times), Sright(1)…Sright(times))
Get the subsets of LBPCode1and LBPCode2 corresponding to S.
Take the subsets as the new LBPCodes.
generate the BBM of new LBPCodes using definition 1 and 2.

By means of Algorithm 1, the main idea of generating the PBBM of a certain individual with many samples is described as follows. First we use algorithm 1 to get the BBM for each pair of samples. Then we take these BBMs as new LBPCodes. Finally use these new LBPCodes to get the PBBM according to definition 1 and 2. The pseudo-code of getting the PBBM of a certain individual is summarized in Algorithm 2.

**Algorithm 2.** GetPBBM.

**INPUT:** LBPCode_1_…..LBPCode_m_ extracted from m samples captured by a certain individual.
**OUTPUT:** PBBM of the certain individual.
**Algorithm:**
For n=1 to $C_{m}^{2}$ do
sampling two samples with non-replacement;
get BBM(n) by invoking GetBBM_twosamples.
endfor
take these BBMs as new LBPCodes.
use these new LBPCodes to generate PBBM according to Definition 1 and 2.

## 4. The Proposed Method

**Figure 3.**
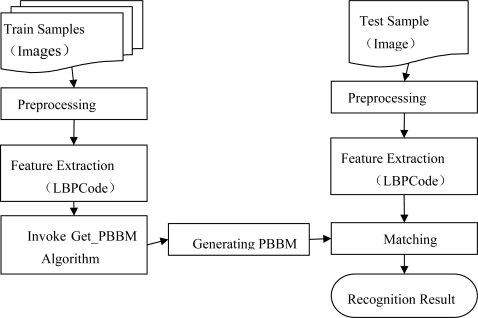
Framework of the proposed method.

Based on the mentioned algorithms 1 and 2, we propose a finger vein recognition method based on PBBM. It mainly involves two stages: a training stage and a recognition stage. The training stage aims to generate the PBBM for each individual, which includes preprocessing, feature extraction, invoking Get_PBBM algorithm and finally generating the PBBM. In the recognition stage, we first preprocess the test sample, and then extract the LBPCode, and next compute the similarity between the LBPCode and the PBBM of a certain individual, finally obtaining a recognition result with a given threshold. The framework of the proposed method is demonstrated in Figure 3.

### 4.1. Preprocessing

The preprocessing operation mainly refers to image gray processing, ROI extraction, size normalization and gray normalization.

**Figure 4.**
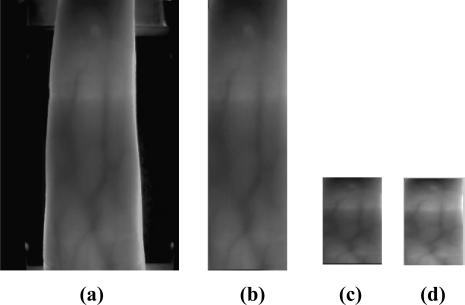
Examples of preprocessing.

**Image gray processing**: the original image (an example is shown in Figure 4(a)) captured by the device in this paper is a 24-bit color image with a size of 320 × 240. In order to reduce the computational complexity, we transform the original image to an 8-bit gray image based on the gray-scale equation *Y* = *R* × 0.299 + *G* × 0.588 + *B* × 0.114, where *R*, *G*, and *B* denote the value of red, green, and blue primaries.

**ROI extraction**: As the background of finger vein region might include noise, we employ an edge-detection method to segment the finger vein region from the gray-scale image. A Sobel operator with a 3×3 mask $\left[\begin{array}{ccc} -1 & 0 & 1\\ -2 & 0 & 2\\ -1 & 0 & 1 \end{array}\right]$ is used for detecting the edges of fingers. The width of the finger region can be obtained based on the maximum and minimum abscissa values of the finger profile, and the height of the finger region is similarly detected. A rectangle region can be captured based on the width and height. This rectangle region is called ROI (as shown in Figure 4(b)).

**Size normalization:** The size of the ROI is different from image to image due to personal factors such as different finger size and changing location. Therefore it is necessary to normalize the ROI region to the same size before feature extraction. We use the bilinear interpolation for size normalization in this paper, and the size of the normalized ROI is set to be 96 × 64 (as show in Figure 4(c)).

**Gray normalization**: In order to extract efficient features, gray normalization is used to obtain a uniform gray distribution (as shown in Figure 4(d)). In this paper, we use:

(5) $$p(i,j)=\frac{p^{\prime }(i,j)-G_{1}}{G_{2}-G_{1}}$$

where *p*’(*i*, *j*) is the gray value of pixel (i, j) of original image, *G_1_* is the minimum gray value of original image, and *G_2_* is the maximum gray value of original image.

### 4.2. Matching

The proposed method measures similarities between the extracted LBPCode and the PBBM of a certain individual. The matching score is estimated as follows:

(6) $$\text{Matching Score}=1-\frac{\left\|\text{PBBM}⊗\text{LBPCode}\right\|}{\text{Length of PBBM}}$$

In Equation (6), ⊗ is a Boolean exclusive-OR operator between two binary patterns; the length of PBBM is the numbers of the Best Bit of a certain individual. Note that not every bit of LBPCode, but only the bits which have the same location as in the PBBM are operated on by the Boolean exclusive-OR operator.

## 5. Experimental Results

### 5.1. The Experimental Database

**Figure 5.**
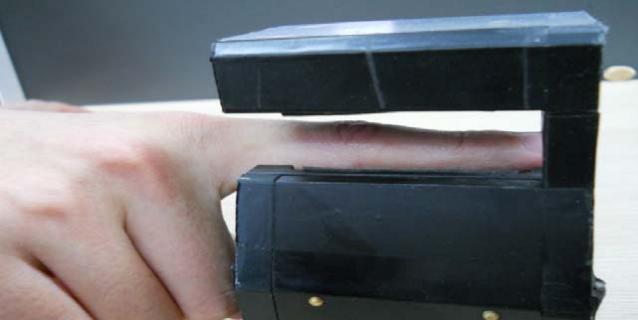
The data capture device.

The experiments were conducted using our finger vein database which is collected from 106 individuals (including 86 males and 20 females, Asian race) index fingers of right hand, where each index finger contributes 14 finger vein images. Each individual participated in two sessions, separated by two weeks (14 days). In each session, seven samples were collected from right hand of each individual. The age of the participants was between 19 and 60 years and their occupations included university students, professors, and workers at our school. The capture device was manufactured by the Joint Lab for Intelligent Computing and Intelligent System of Wuhan University, China. The capture device mainly consists of a near-infrared light source, lens, light filter, and image capture equipment. Vein patterns cannot be observed using normal, visible rays of light since they are beneath the skin’s surface. However, vein patterns can be viewed through an image sensor which is sensitive to near-infrared light (wavelengths between 700 and 1,000 nanometers), because near-infrared light passes through human body tissues and is blocked by pigments such as hemoglobin or melanin. To obtain a stable finger-vein pattern, our light source uses a near-infrared light source with wavelength of 890 μm, the image capture part uses a near-infrared CCD camera with a wavelength of 900 μm. A groove in the shell of the device is used to guide the finger orientation, and the capture device is illustrated in Figure 5.

**Figure 6.**
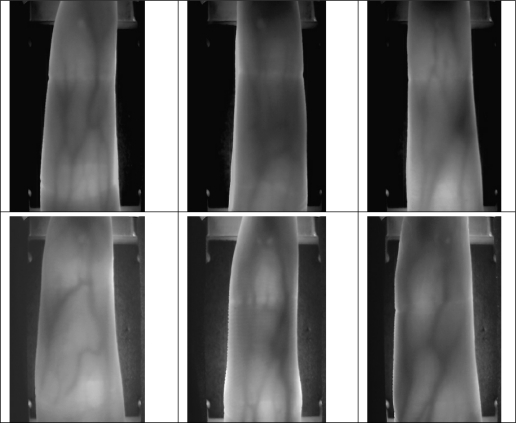
Sample finger vein images.

The original spatial resolution of the data is 320 × 240, After ROI extraction and Size Normalization, the size of the region used for feature extraction is reduced to 96 × 64. Samples collected from the same finger belong to the same class. Therefore, there are 106 classes, where each class contains 14 samples in our database. Some typical finger vein images are shown in Figure 6.

### 5.2. The Experimental Settings

All the experiments are implemented in MATLAB, and conducted on a PC with 2.4G CPU and 2G memory. Considering the misalignment between images from the same individual is not obvious, we set *moving step* as 1 pixel, and *moving times* as 4. In this paper, three experiments are designed to multilaterally evaluate the proposed method: (a) Experiment 1 evaluates performance of the proposed method in verification mode and identification mode respectively, and compares with the LBP-based method. (b) Experiment 2 evaluates the effect on recognition performance when using different number of samples to generate the PBBM. (c) Experiment 3 evaluates the robustness of the PBBM.

### 5.3. Experiment 1

First the average processing times were measured, as shown in Table 2. The average preprocessing time *per* image was 53 ms; the average feature extraction (LBPCode) time *per* image was 0.9 ms; the average PBBM training time for 4 samples was 423 ms, and the average matching time between the PBBM and the LBPCode was 16 ms. Although the average training time is somewhat time-consuming, the training process can be done off-line. In a word, from Table 2, we can see that the proposed method can be used in real-time.

We performed two types of experiments on the established database: verification mode and identification mode. In the verification mode, the class of the input finger vein (test sample) is known, and each test sample is matched with the PBBMs of every class. A successful matching is called intraclass matching or genuine, if the test sample and PBBM are from the same class. Otherwise, the unsuccessful matching is called interclass matching or imposter. In the experiments, we use the first four samples of each class in the database to generate the PBBM and use the other 10 as test samples. Consequently, there are 1,060 (106 × 10) intraclass matchings and 111,300 (106 × 10 × 105) interclass matchings in total. In this paper, the performance of a system is evaluated by the EER (equal error rate), the FRR (false rejection rate) at zero FAR (false acceptance rate) and the FAR at zero FRR. The EER is the error rate when the FRR equals the FAR and therefore suited for measuring the overall performance of biometrics systems because the FRR and FAR are treated equally. On the other hand, the FRR at-zero-FAR is suited for high security systems, as in those systems, false acceptance errors are much more critical than false rejection errors. On the contrary, the FAR at zero FRR shows the acceptance rate of impostors when none genuine rejected is desired.

**Figure 7.**
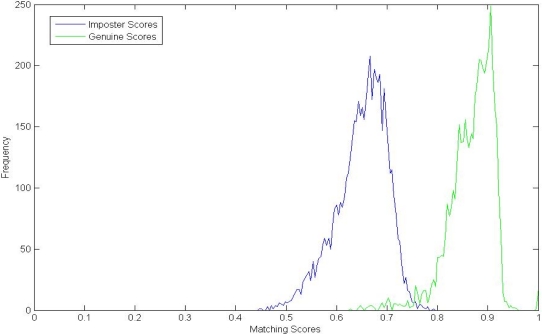
Genuine and imposter matching score distributions by LBP.

**Figure 8.**
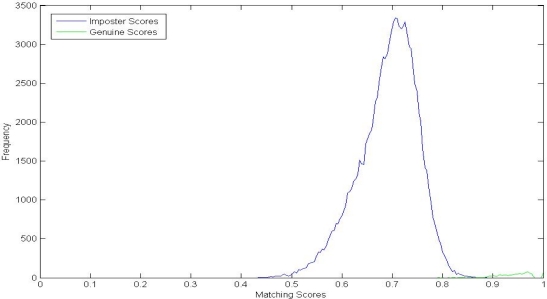
Genuine and imposter matching score distributions by PBBM.

We compare the proposed method with the LBP-based method in [27], Genuine and imposter matching score distributions of these two methods are shown in Figures 7 and 8, respectively. From Figure 7, we can see that the genuine and imposter match scores by LBP are overlapped between 0.6 and 0.8. Otherwise, the genuine matching scores by PBBM are mainly between 0.85 and 1.00, and the imposter matching scores are mainly between 0.55 and 0.80.

**Figure 9.**
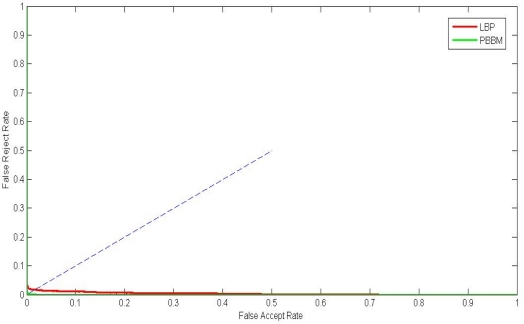
ROC curves by different method.

The ROC curves are shown in Figure 9. The ERR, FRR at-zero-FAR and FAR at-zero-FRR values are listed in Table 3. From Figure 9 and Table 3, we can see that the proposed method achieves a much lower EER than the LBP-based method. This indicates that the PBBM is beyond suitable features which truly reflect the characteristics of a certain individual. Besides, it can remove the noisy bits. Because the PBBMs of every individual are different from each other, the PBBM can better illustrate the differences between the individuals.

To verify the robustness of the PBBM trained by samples acquired in different sessions, we randomly select four samples of each class in the database to generate PBBM and used the other 10 as test samples. We repeat this process ten times, and the statistical data of the verification performance (EER) is shown in Table 4. From Table 4, we can see that the PBBM is robust for training and testing samples acquired from different sessions.

**Figure 10.**
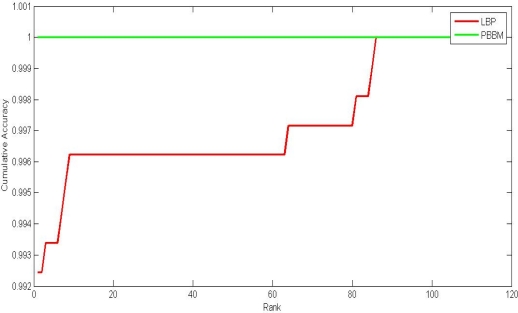
Cumulative match curves by different methods.

The experiments of closed-set identification (identification is performed only for individuals who are present in the enrollment database) were also conducted. In the identification mode, we do not know the class of the input finger vein, but want to identify which class it belongs to. Like the verification mode, we use the first four samples of each class in the database to generate the PBBM and use the other ten as test samples. Therefore, there are 106 templates, and 1,060 (106 × 10) probes in total. The probes were matched with all the templates models. For each probe, the matching results were ordered according to the matching scores. Then, we can get the cumulative match curves as shown in Figure 10. The cumulative matching performance, rank-one recognition rate, and lowest rank of perfect recognition (*i.e.*, the lowest rank when the recognition rate reaches 100%) are listed in Table 5. From the experimental results, we can see that the performance of the proposed method is much better than that of the LBP-based methods. The rank-one recognition rate of the proposed method was 100%, indicating that all probes can be identified properly.

### 5.4. Experiment 2

As mentioned above, a certain number of samples are needed to generate the PBBM for each individual. This experiment evaluates the effect on recognition performance when using different numbers of samples to generate a PBBM. For the sake of justice, we measure by fixing the number of testing samples and adjusting the number of training samples to generate the corresponding PBBMs. We set the last six as testing samples of each class in the established database, so that the number of training samples is between 1 and 8. It should be pointed out that when taking only one as the training sample, we regard each bit of its LBPCode as the Best Bit. Therefore, the LBP-based method can be taken as a special case of the proposed method.

**Figure 11.**
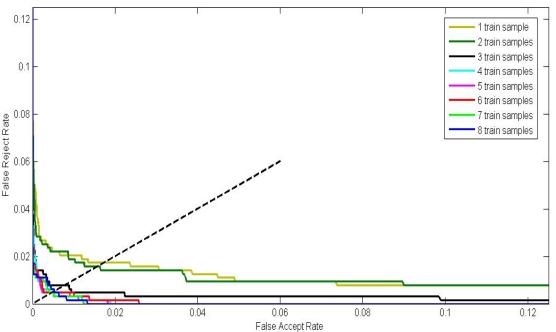
ROC curves by different number of training samples.

The ROC curves of PBBMs trained by different samples are shown in Figure 11, The ERR, FRR at-zero-FAR and FAR at-zero-FRR values are listed in Table 6. From Figure 11 and Table 6, we can see that better performance is achieved with an increasing number of training samples. When the number of training samples reaches 4, the performance promotion is limited. It indicates that we can only use four training samples of each class to generate a competitive PBBM.

**Figure 12.**
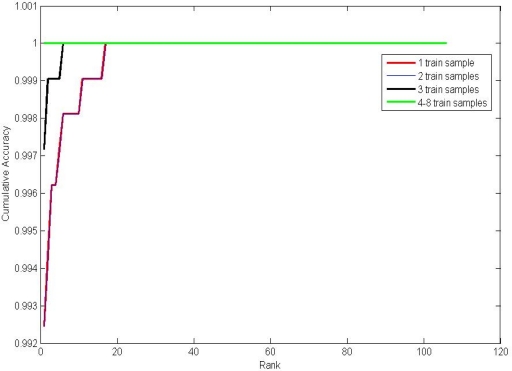
Cumulative match curves by different number of training samples.

We also conduct experiments on the identification mode. Figure 12 and Table 7 illustrate the expriment results. Apprently, when the number of training samples reaches 4, the rank-one recognition rate can achieve 100%.

### 5.5. Experiment 3

From experiments 1 and 2, we can see that the PBBM achieved promising results. The basis supporting our study is that the PBBM must be robust and stable. In this experiment, we will discuss the robustness of PBBM, mainly including whether and how the PBBM is convergent when the number of training samples increases. In this paper, we evaluate the robustness with the percentage of the number of bits of the PBBM relative to the number of bits of its original LBPcode. Table 8 shows the statistical data of the percentage by different number of train samples using Minimum, Maximum, Mean, and Variance values. The Mean value can evaluate the reliability of the PBBM, and the Variance value can evaluate the stability of the PBBM. The Mean value of the percentage is decreased when the number of training samples increases. As mentioned above, when the number of training samples reaches 4, the recognition performance is promising. The corresponding mean percentage is 0.7986; this means that the number of Best Bits of each class is sufficient for matching, and the matching result is reliable. As a whole, the variance value of the percentage is very small, which indicates the good stability of the PBBM.

**Figure 13.**
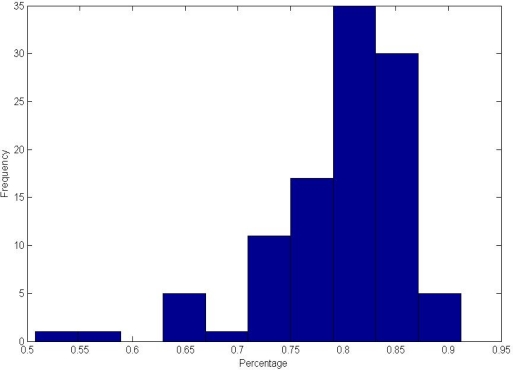
Histograms of the percentage by four training samples.

The histogram of the percentage of 106 classes for four training samples is shown in Figure 13. From Figure 13, we can see that the majority of percentages are near 0.80. In a word, the robustness of the PBBM is very high, and the recognition results are credible.

If the percentage of the number of bits of the PBBM relative to the number of bits of its original LBPcode is very low, it means that the samples of this individual have low image quality, e.g., there may be a large number of noisy bits in the corresponding LBPCode, so we can use the percentage or size of the PBBM to evaluate the image quality of samples. For example, we acquire four samples of a certain individual during the enrollment process, and use these samples to generate his/her PBBM. If the size of its PBBM is too small, this individual can be rejected for enrolment.

## 6. Conclusions and Future Work

This paper presents a novel finger vein recognition method base on a PBBM. The experimental results show the superior performance of our method in comparison with the LBP-based method. The advantages of PBBM can be summarized as follows: (1) PBBM effectively removes noisy bits. (2) PBBMs are different from individual to individual, thus PBBMs can be regarded as a kind of personalized feature that reflects the differences between each individuals remarkably. (3) The PBBM is highly robust and reliable.

It should be pointed out that PBBM is a general framework for binary pattern based recognition. Besides LBP, we can use LDP, LLBP and other binary code to generate the corresponding PBBM, and this will be the subject of our future work. In addition, our database has a limited number of individuals, so we plan to apply PBBM to large-scale real-world databases.

## Figures and Tables

**Table 1.** Methods for personal authentication using finger vein recognition.

**References**	**Method**	**Database fingers × samples per each**	**Performance**
[6]	line-tracking	339 × 2 images	EER: 0.145%
[8]	manifold learning	164 × 70 images	EER: 0.8%
[9]	Radon transform	50 × 10 images	GAR: 99.2%.
[11]	Minutiae points	50 × 10 images	EER: 0.761%
[12]	mean curvature	125 × 9 images	EER : 0.25%
[13]	Maximum Curvature Points	7 × 14 images	GAR: 95%
[14]	wide line detector	10,140 × 5 images	EER: 0.87%
[15]	minutia points	816 × 10 images	EER: 1.91%
[20]	curvelets and local interconnection structure	400 × 8 images	EER: 0.128%
[21]	Local moment, middleological,Vein-shape	162 × 10 images	GAR: 99%
[22]	2-D Gabor filters	300 × 5 images	GAR: 99.31%.

**Table 2.** The average processing times.

**Preprocessing**	**Feature extraction**	**Training PBBM**	**Matching**
53 ms	0.9 ms	423 ms	16 ms

**Table 3.** verification performance by different methods.

	**EER**	**FRR at-zero-FAR**	**FAR at-zero-FRR**
LBP method	0.018	0.0497	0.7182
Proposed method	0.0038	0.0199	0.0433

**Table 4.** the statistical data of EER by random sampling.

**Minimum**	**Maximum**	**Mean**	**Variance**
0.0013	0.0066	0.0041	3.027 × 10^−6^

**Table 5.** Identification performance by different methods.

	**rank-one recognition rate**	**lowest rank of perfect recognition**
LBP based method	99.25%	86
Proposed method	100%	1

**Table 6.** Verification of performance with different numbers of training samples.

	**EER**	**FRR at-zero-FAR**	**FAR at-zero-FRR**
1 training sample	0.0173	0.1038	0.6972
2 training samples	0.0154	0.1258	0.7073
3 training samples	0.0079	0.0865	0.2274
4 training samples	0.0047	0.0189	0.0692
5 training samples	0.0047	0.0183	0.0566
6 training samples	0.0047	0.0259	0.0629
7 training samples	0.0047	0.0124	0.0362
8 training samples	0.0053	0.0133	0.02672

**Table 7.** Identification performance by different number of training samples.

	**Rank-one recognition rate**	**Lowest rank of perfect recognition rate**
1 training sample	99.06%	17
2 training samples	99.06%	17
3 training samples	99.72%	6
4 training samples	100%	1
5 training samples	100%	1
6 training samples	100%	1
7 training samples	100%	1
8 training samples	100%	1

**Table 8.** Statistical data of the percentage by different number of training samples.

	**Minimum**	**Maximum**	**Mean**	**Variance**	**EER**
1 training sample	1.0000	1.0000	1.0000	0.0000	0.0173
2 training samples	0.8158	1.0000	0.9021	0.0007	0.0154
3 training samples	0.5820	0.9118	0.8395	0.0025	0.0079
4 training samples	0.5078	0.9118	0.7986	0.0042	0.0047
5 training samples	0.4729	0.8963	0.77	0.0052	0.0047
6 training samples	0.4562	0.8963	0.7508	0.0056	0.0047
7 training samples	0.4052	0.8912	0.7055	0.0061	0.0047
8 training samples	0.3891	0.8133	0.678	0.0061	0.0053
